# Primary Large Broad Ligament Fibroid: A Challenge in Surgical Practice

**DOI:** 10.7759/cureus.51415

**Published:** 2023-12-31

**Authors:** Efthymia Thanasa, Anna Thanasa, Vasiliki Grapsidi, Emmanouil M Xydias, Gerasimos Kontogeorgis, Ioannis-Rafail Antoniou, Evangelos Kamaretsos, Apostolos C Ziogas, Ioannis Paraoulakis, Ioannis Thanasas

**Affiliations:** 1 Department of Health Sciences, Medical School, Aristotle University of Thessaloniki, Thessaloniki, GRC; 2 Department of Obstetrics and Gynecology, General Hospital of Trikala, Trikala, GRC; 3 Department of Obstetrics and Gynecology, EmbryoClinic IVF Unit, Thessaloniki, GRC; 4 Department of Obstetrics and Gynecology, University of Thessaly, Larissa, GRC

**Keywords:** primary broad ligament fibroid, computed tomography, magnetic resonance imaging, ureteral stents, surgical treatment, ultrasonic surgical scalpel, intraoperative complications, case report

## Abstract

Primary broad ligament fibroids, whose surgical treatment is challenging, are extremely rare. Our case concerns the surgical treatment of a large broad ligament fibroid. A 48-year-old patient, asymptomatic and with a medical history of uterine leiomyomas, came to the gynecology outpatient clinic to undergo a routine gynecological examination. On bimanual pelvic examination, the presence of a painless palpable pelvic mass was found, without being able to clinically demarcate it. Computed tomography imaging confirmed the clinical suspicion of a pelvic mass. The pelvic mass was more consistent with the subserosal pedunculated fibroid of the uterine corpus, but the preoperative diagnosis of adnexal mass cannot be excluded. It was decided to surgical treatment of the patient with a total hysterectomy and bilateral salpingectomy-oophorectomy. Intraoperatively, the presence of a large intraligamental mass was detected. The uterus, cervix, and ovaries were normal but displaced by the tumor. After resection of the leiomyoma from the broad ligament, where it was not found to be connected to a vascular pedicle from the lateral wall of the uterine corpus or the cervix, total hysterectomy and bilateral salpingectomy-oophorectomy were performed, due to the necessary resection of the right fallopian tube and ovary and the extensive injuries in the area. The postoperative course was uneventful. In this paper, following the case presentation, a brief review of primary broad ligament fibroids is presented, emphasizing the significance of comprehensive preoperative planning in the challenging intraoperative management of these patients, who have an increased risk of intraoperative complications.

## Introduction

Fibroids or leiomyomas are common neoplasms of the uterus [1]. Much more common are leiomyomas of the uterine corpus and cervix (subserosal, intramural, submucosal) compared to parasitic extrauterine leiomyomas. Parasitic leiomyomas may grow in the broad ligament or, less frequently, in other anatomical structures where smooth muscle is present, such as the round ligament, the utero-ovarian ligament, and the ovaries. The broad ligament is composed of a double-layer fold of the visceral peritoneum, enveloping the uterus and extending bilaterally from the lateral walls of the uterine corpus to the lateral pelvic sidewalls [2]. The broad ligament is the most common location of extrauterine leiomyoma (broad ligament fibroids). Primary broad ligament fibroids originate from smooth muscle fibers contained in the extraperitoneal tissue between the leaves of the broad ligament, along with connective tissue and neurovascular components. It is essential to differentiate broad ligament fibroids from intraligamental leiomyomas. Intraligamental leiomyomas are pedunculated tumors that originate in the lateral wall of the uterine corpus or cervix and grow within the leaves of the broad ligament. Primary broad ligament fibroid, as observed in our patient, is not attached to a vascular pedicle, neither to the uterus nor to the cervix [3].

On the occasion of our case, it is emphasized that, despite the rarity of large primary broad ligament fibroid, comprehensive preoperative planning of the patient and the surgical skills of the medical team are essential for achieving the optimal outcome of the surgery and the successful management of potential intraoperative complications associated with these procedures.

## Case presentation

Our 48-year-old patient came to the gynecology outpatient clinic of the General Hospital of Trikala to undergo a routine gynecological examination. The patient had a medical history of uterine fibroids. Ten years ago, she had undergone enucleation of a leiomyoma with laparotomy. The cervical smear test (Pap test) was negative for malignancy. Subsequent transvaginal ultrasound, conducted after the cervical smear test, revealed the presence of a small uterine leiomyoma. For the last four years, due to the COVID-19 pandemic, our patient did not undergo a routine gynecological check-up. Her medical history revealed that the patient had a regular menstrual cycle and had given birth to two children by cesarean section. She reported no intestinal disorders or weight loss. She occasionally complained of frequent urination without accompanying urinary tract infection. Medical disorders such as hypothyroidism and arterial hypertension were reported, and well regulated with appropriate medication. On gynecological examination, the cervix was free of abnormal findings. However, the examination identified the presence of a painless palpable pelvic mass, challenging to clinically demarcate due to the patient's increased weight (BMI = 31).

**Figure 1:**
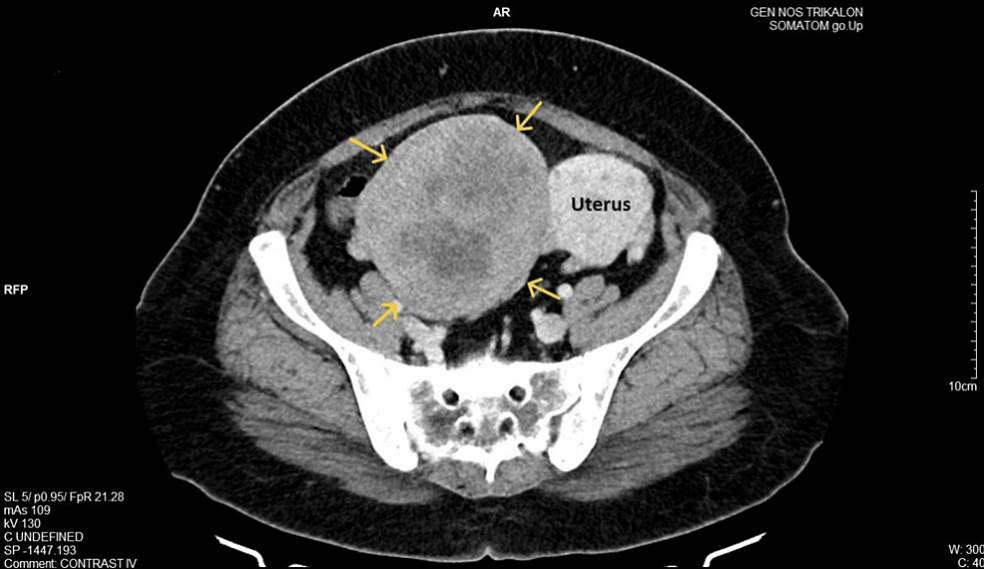
CT imaging of primary broad ligament fibroid The image depicts the presence of a large exophytic mass (yellow arrows), which appears to be attached to the right lateral wall of the uterus and occupies a substantial part of the peritoneal cavity

The transvaginal ultrasound was not diagnostic: a large solid echogenic mass was found, which occupied the pouch of Douglas and extended to the right of the uterus, but the upper margins could not be imaged. Similarly, transabdominal ultrasound was nondiagnostic because of the patient's increased weight. A computed tomography (CT) scan revealed an enormous soft tissue exophytic mass, measuring 10.6 x 11 x 16 cm, in direct contact with the right lateral wall of the uterus, from which it appeared to be arising (Figure 1).

The lesion seemed to cause compression effects on the upper bladder wall and the body of the uterus, displacing it to the left of the midline. The CT findings were more consistent with a subserosal pedunculated fibroid of the uterine corpus, but the preoperative diagnosis of adnexal mass cannot be ruled out. MRI was not available at the hospital. The patient's blood tests were normal (Table 1). Tumor markers (CEA, Ca125, Ca15-3, Ca19-9) were within the normal range.

**Table 1:** Preoperative and postoperative laboratory analysis of a patient with primary broad ligament fibroid Ht – Hematocrit, Hb – Hemoglobin, PLT – Platelets, WBC – White Blood Cells, NEUT – Neutral, APTT – Activated Partial Thromboplastin Time, INR – International Normalized Ratio, FIB – Fibrinogen, U – Urea, Cr – Creatinine

Laboratory tests	Preoperatively	1st postoperative day after laparotomy	4th postoperative day after laparotomy	Normal laboratory values
Ht	39.9%	35.9%	36.2%	37.7 – 49.7%
Hb	13.6 gr/dl	11.9 gr/dl	12.1 gr/dl	11.8 – 17.8 gr/dl
PLT	292 x 10^3^/ml	205 x 10^3^/ml	224 x 10^3^/ml	150 – 350 x 10^3^/ml
WBC	8.8 x 10^3^/ml	18.9 x 10^3^/ml	7.3 x 10^3^/ml	4 – 10.8 x 10^3^/ml
NEUT	73.3%	89.3%	65.4%	40 – 75%
APTT	33.2 sec	34.2 sec	33.1 sec	24.0 – 35.0 sec
INR	0.94	1.03	0.98	0.8 – 1.2
FIB	291 mg/dl	265 mg/dl	255 mg/dl	200 – 400 mg/dl
U	29 mg/dl	35 mg/dl	22 mg/dl	10 – 50 mg/dl
Cr	0.82 mg/dl	0.87 mg/dl	0.51 mg/dl	0.40 – 1.10 mg/dl

**Figure 2:**
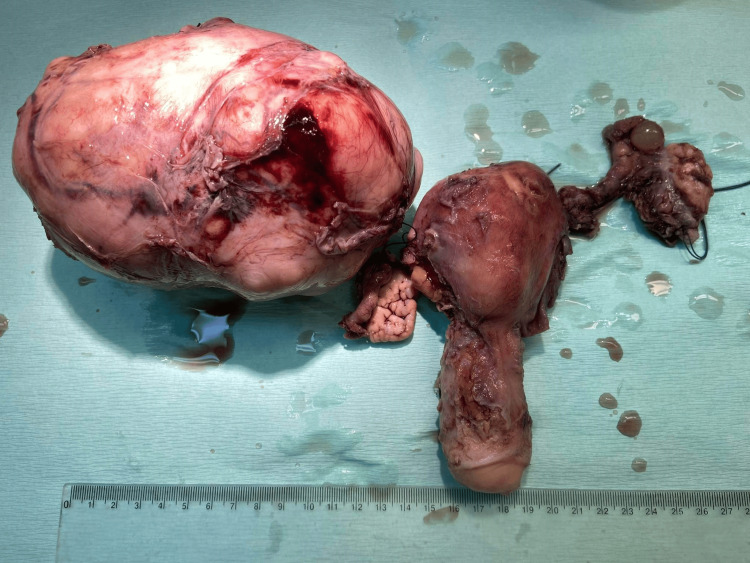
Surgical specimen of primary broad ligament leiomyoma No vascular pedicle is visible connecting the leiomyoma to the wall of the uterus or cervix

Following a thorough consultation with the patient, it was decided to proceed with an exploratory laparotomy. The patient consented that a total hysterectomy with bilateral salpingectomy-oophorectomy may be necessary. Preoperative ureteral stent placement was feasible only in the left ureter. Intraoperatively, the presence of a large intraligamental tumor, with hard consistency was identified in the right parametrium, causing significant right ovarian misplacement to the mid-abdominal level. There was no observable invasion of the visceral peritoneum by the tumor. The uterus, cervix, and adnexa appeared normal (Figure 2).

Following the dissection of the anterior leaf of the broad ligament, the fibroid was successfully removed from the intraligamental space without identifying any attachment to a vascular pedicle from the lateral wall of the uterine corpus or the cervix. Dissection of the leiomyoma from the leaves of the broad ligament was performed with utmost care to prevent injury to the ureters, bladder, and the large vessels and their branches passing through the anatomical area. The surgery was completed with the resection of the uterus and the fallopian tubes-ovaries, without significant blood loss (Table 1).

**Figure 3:**
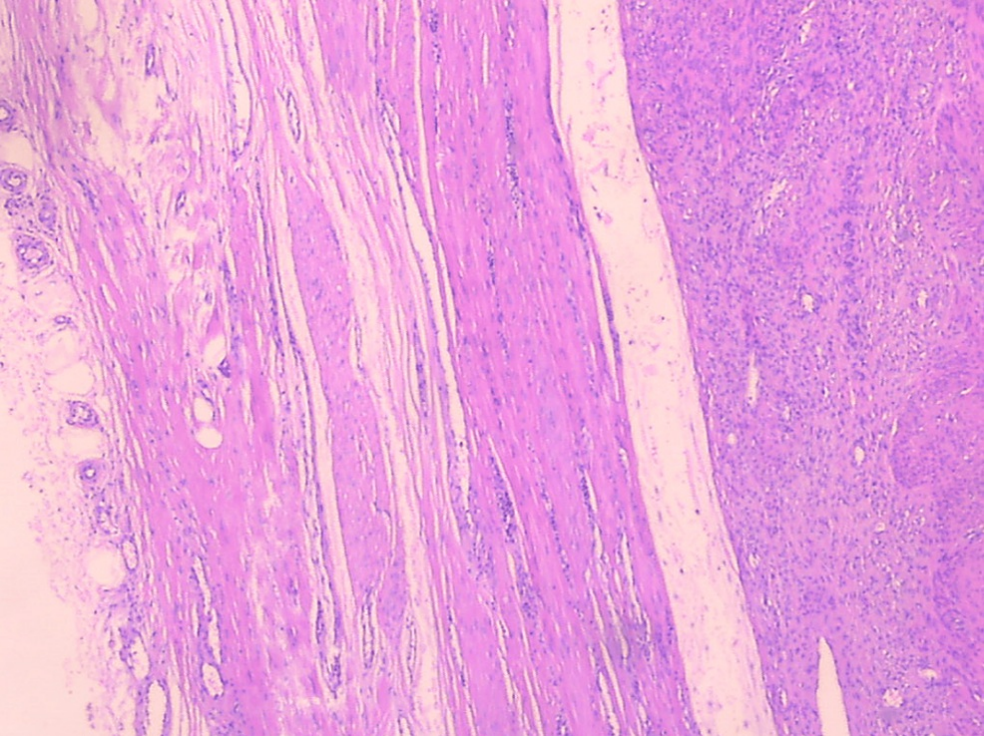
Histological image of primary broad ligament fibroid The visualization of the smooth circumscribed borders of the tumor is evident

**Figure 4:**
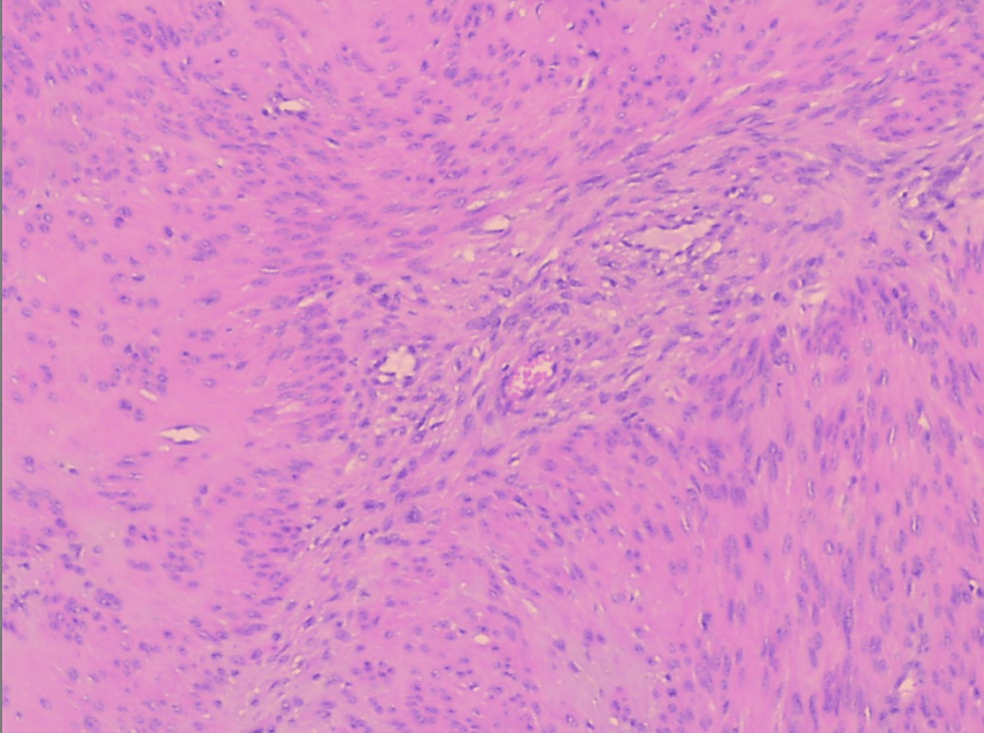
Histological image of primary broad ligament fibroid A fascicular cell growth pattern with eosinophilic cytoplasm without atypia and mitoses is evident

Histological examination of the surgical specimen confirmed the diagnosis of primary broad ligament fibroid. Macroscopically, an oval-shaped tumor with a maximum diameter of 17 cm with a smooth outer surface entirely covered by serosa was found, with no indications of tumor attachment to the uterine corpus or cervix (Figure 2). Microscopic examination revealed mild to moderate cellularity, scarce mitoses and absence of necrosis and cellular atypia. Additionally, vitrification of the stroma and focal myxoid and cystic degeneration were observed in certain areas (Figure 3, Figure 4).

Following an uneventful postoperative course, the patient, hemodynamically stable (Table 1) was discharged from the clinic on the fifth postoperative day. The patient was put on hormone replacement therapy.

## Discussion

Proper preoperative planning for patients with broad ligament fibroid should be included in the essential requirements for the successful outcome of the selected surgery. It is estimated that the larger the size of the primary broad ligament fibroid, the more extensive the preoperative planning of the myomectomy or total hysterectomy selected for the treatment of these patients should be [4]. Preoperative differential diagnosis of broad ligament fibroid by ultrasound, CT, or MRI imaging is difficult. To ascertain that the leiomyoma primarily originates from the broad ligament (true broad ligament fibroid), it is crucial to ensure, based on imaging, that it has no origin from the uterus or adnexa. It is currently considered that MRI is deemed more significant than ultrasound and CT in differentiating broad ligament fibroids from subserosal leiomyomas of the uterus or adnexal masses [5,6]. It is not surprising that both ultrasound and CT scans failed to establish the diagnosis of broad ligament fibroid in our patient. Also, preoperative bilateral ureteral stent placement by the urologist is considered necessary to prevent the ureters from potential intraoperative damage. After careful dissection of the broad ligament fibroid from the leaves of the broad ligament and the parametrium, ureteral stents facilitate intraoperative ureteral detection and safe hemostasis with minimal risk of intraoperative damage to the anatomical area [7]. In our patient, the unsuccessful placement of a ureteral stent in the right ureter further increased the degree of difficulty of the surgery. The careful separation of the parametrial tissues from the tumor and the experience of the surgical team could and did preserve the integrity of the ureter. Also, screening for deep venous thrombosis should not be omitted in patients with large broad ligament fibroids. Finally, consultation with the hospital's blood bank and preoperative preparation of packed red blood cells to deal with potential massive hemorrhage requiring blood transfusion is considered essential [8].

The surgical treatment of broad ligament fibroid is a challenge in clinical practice [9]. Beyond meticulous preoperative preparation of the patient, a profound understanding of the anatomy of the bladder, ureters, and other pelvic structures displaced by the presence of the leiomyoma is imperative. Moreover, the skills of the experienced surgical team called upon to treat patients with large broad ligament fibroid is included in the necessary requirements for a safe and successful outcome of myomectomy or total hysterectomy via laparotomy or laparoscopy [9]. It should be highlighted that, regardless of the patient's desire to preserve or not preserve fertility, myomectomy is necessary before performing a total hysterectomy, particularly in cases involving large intraligamental tumors. It is understood that, following the resection of the broad ligament fibroid, there is a partial restoration of the demarcation of pelvic anatomical structures displaced by the tumor. This restoration facilitates the subsequent resection of the uterus and fallopian tubes-ovaries with increased convenience and safety [10]. Similarly, in our patient, although preoperatively it was decided to resect the uterus with bilateral salpingectomy-oophorectomy, intraoperatively it was chosen to resect the intraligamental leiomyoma first before performing a total hysterectomy. The myomectomy significantly contributed to an improved surgical plane and facilitated surgical maneuvers. Moreover, on the occasion of the surgical treatment of our case, we highlight the dissection and ligation of the suspensory ligament as the second demarcation point of the incision on the broad ligament, following the dissection and ligation of the ipsilateral round ligament (first demarcation point). We believe that this technique, combined with the use of an ultrasonic surgical scalpel, facilitates the resection of leiomyoma from the parametrium, minimizes intraoperative blood loss, and reduces the potential thermal damage to the ureters, bladder, and intestine. In all cases where intra-operative hemorrhage is observed to be increased, immediate blood transfusion is recommended. It is essential to consider the risk of underestimating intraoperative blood loss in such cases.

## Conclusions

Primary broad ligament leiomyomas are extremely rare. Proper preoperative preparation and an appropriate therapeutic approach by an experienced surgical team are crucial to ensuring an optimal surgical outcome and minimizing the increased risk of intraoperative complications. Myomectomy should be prioritized as the initial surgical step, even in cases where a total hysterectomy with bilateral salpingectomy-oophorectomy has been preoperatively decided. Additionally, it is recommended to perform dissection and ligation of the suspensory ligament as a secondary point of demarcation for the incision on the broad ligament. The use of a surgical ultrasonic scalpel facilitates surgical maneuvers and reduces the risk of intraoperative complications.
